# Evaluation of Priming Efficiency of Forskolin in Tissue-Specific Human Mesenchymal Stem Cells into Dopaminergic Neurons: An In Vitro Comparative Study

**DOI:** 10.3390/cells9092058

**Published:** 2020-09-09

**Authors:** Manisha Singh, Pardeep Kumar Vaishnav, Amit Kumar Dinda, Sujata Mohanty

**Affiliations:** 1 Stem Cell Facility (DBT-Centre of Excellence for Stem Cell Research), All India Institute of Medical Sciences, New Delhi 110029, India; msingh37@jhmi.edu; 2 Dr. Solomon H. Snyder Department of Neurosciences, Johns Hopkins University, Baltimore, MA 21218, USA; 3 Electron Microscopy, All India Institute of Medical Sciences, New Delhi 110029, India; pardeepvaishnav@yahoo.com; 4 Department of Pathology, All India Institute of Medical Sciences, New Delhi 110029, India; amit_dinda@aiims.edu

**Keywords:** forskolin, dopaminergic neurons, mesenchymal stem cells, calcium ion imaging, scanning electron microscopy

## Abstract

Background: Human mesenchymal stem cells (hMSC) can be derived from various tissue sources and differentiated into dopaminergic (DAergic) neurons using various types of inducers. There are several strategies that have been reported to generate functional dopaminergic neuronal cells from hMSCs in the most efficient manner possible. However, this area is still under extensive research. In this study, we aim to compare hMSCs derived from bone marrow (BM), adipose tissue (AD) and dental pulp (DP) to generate functional dopaminergic neurons, using FGF2 and forskolin. Post-differentiation, multiple factors were used to characterize the cells at morphological, morphometric, ultra-structural, mRNA and protein levels for various markers (Nestin, NF, MAP2, Tuj1, TH, DAT, PitX3, Ngn2, Kv4.2, SCN5A). Cells’ functionality was studied by calcium ion imaging, along with the amount of dopamine secreted by the cells in the culture medium. Results: Data analysis revealed that forskolin has comparable effect on BM- and AD-derived MSC (28.43% and 29.46% DAergic neurons, respectively), whereas DP-MSC (42.78 ± 1.248% DAergic neurons) show better outcome in terms of efficient generation of DAergic neuronal cells, expression of neuronal associated markers, dopamine release and calcium ion efflux. Ultra-structural studies by SEM and TEM also revealed a substantial change in both cellular morphology and composition of cellular organelles. It was observed that AD-MSCs showed the best neuronal features, at morphological, gene, and protein levels upon induction with the above-mentioned induction cocktail. Conclusion: It may be concluded that a combination of FGF2 and forskolin yields functionally active dopaminergic neuronal cells in vitro, with highest percentage of the same from AD-MSCs, as compared to that in BM-MSCs and DP-MSCs. The outcomes and comparative evaluation provide a substantial platform for further studies on molecular pathways involved in the process of DAergic neurogenesis in individual cases.

## 1. Background

Forskolin (FSK) is a diterpene adenylate cyclase activator and is commonly used to increase the level of cyclic AMP (cAMP) and cAMP-responsive element-binding (*CREB*), a downstream target of Forskolin, has been reported to regulate neuronal specification and promote axonal regeneration [1]. It enables neurogenin 2 to convert human fibroblasts into cholinergic neurons [2]. There are few recent reports that state the role of cAMP-elevating agents like FSK in neural differentiation of Mesenchymal stem cells. cAMP is the messenger molecule, which acts as intracellular signaling mediator. Its formation is promoted by activation of adenylyl cyclate by FSK, which further occurs after the ligation of G-protein coupled receptors by ligands like prostaglandins, hormones, and pharmacologic agents [3]. This cAMP signaling pathway plays vital role in several cellular mechanisms like cell differentiation, metabolism, and apoptosis. [4].

Neurological disorders are toilsome in management and treatment, as damaged neurons have constrained regenerative potential and the adult nervous tissue exhibit very poor neuro-regeneration. In recent years, stem cell research has set out several modules where neurogenerative diseases can be managed by the transplantation of MSCs. There is extensive evidence at basic, pre-clinical, and clinical stages of research manifesting the regenerative potential of MSCs in various neurodegenerative diseases like Alzheimer’s disease, Parkinson’s disease and Huntington’s disease [5,6,7,8]. Human Mesenchymal Stem Cells (hMSCs) hold enormous potential in the field of regenerative medicine because of several factors associated with them like lack of immunogenicity, tumorogenicity, and immunomodulatory and anti-inflammatory potential. To qualify for MSCs, these cells should be positive for CD105, CD90, CD73, CD29, HLA class I, and negative for HLA class II and CD34/45. They should also be able to show trilineage differentiation potential. [8]. Globally, researchers are venturing into exploring the regenerative potential of MSCs derived from various tissue sources like bone marrow, adipose tissue, dental pulp, Wharton’s jelly, umbilical cord, umbilical cord blood, and amniotic fluid. Yet the most explored tissue source remains bone marrow. Although, there are reports stating the plausible use of adipose tissue-derived MSCs in treating various degenerative diseases, with comparative regenerative efficiency as those derived from bone marrow [6,7,8]. However, the research is still in exploratory mode to establish the actual safety and efficacy of transplantation of MSCs obtained from various tissue sources in various diseases and to establish them for therapy. Use of Mesenchymal stem cells for therapeutic purposes is still under the clinical trial phase [8].

There are very few studies reported to date where forskolin has been used as a neuronal inducer, along with other inducing agents to differentiate hMSCs derived from various tissues into neuronal cells. Levy et al., in 2008, reported the use of forskolin along with a large number of chemicals to induce bone marrow (BM)-derived MSCs into oligodendrocytes. They have used *nestin, NF-68, O4, O1, MBP, S100* and *GFAP* for characterizing the induced cells [9]. Another study reported by Rooney et al., in 2009 states the use of basic fibroblast growth factor, forskolin, ciliary neurotrophic factor and glial-derived neurotrophic factor to induce BM-MSCs into neuronal cells, characterized by expression of *MAP-2ab, NF-M, GFAP* and *TUJ1* markers [10]. Apart from BM-MSCs, Wharton’s jelly and adipose tissue-derived MSCs have also been explored for their neurogenic potential, with forskolin as an important element of the induction media cocktail [11,12].

Most of these studies have used large number inducers, which are mostly chemicals to differentiate hMSCs into cells of neuronal lineage. The use of these chemical inducers is still questionable for translational purpose as their side effects have not been validated yet. The reported papers do not provide sufficient data on morphological, morphometric and ultrastuctural characterization of the in vitro differentiated cells. Most of these studies have explored the potential of hMSCs to differentiate into functional neuronal cells only, but none commented on their efficiency of generation among tissue-specific MSCs. Also, there is no comparative study stating the neuronal differentiation capacities of tissue-specific hMSCs upon induction with forskolin (FSK). This aspect is vital, taking translational aspect of tissue-specific hMSCs into consideration.

Hence, in the current study, we report the in vitro differentiation of human MSCs derived from bone marrow, adipose tissue and dental pulp by using FSK along with FGF2 in minimal concentration to yield dopaminergic neuronal cells. These in vitro differentiated cells were analyzed at morphological, morphometric, transcriptional, translational and ultra-structural levels. Functionality of the cells was also determined by dopamine release assay and calcium ion imaging method, using Fura red-AM ratiometric dye. The study also targeted enumeration of brain cells other than DAergic neuronal cells like acetylcholinergic neurons, serotonergic neurons, Schwann cells and glial cells. Both FGF2 and FSK are FDA approved reagents, hence, they can be implied in clinical set up.

## 2. Methods

The study was commenced after getting ethical clearance from Institutional Committee for Stem Cell Research (IC-SCR) (Ref. No. IC-SCR/37/15(R), dated 7 October 2015), AIIMS, New Delhi. All the methods described in this study were performed in accordance with the relevant guidelines and regulations of the Institution.

### 2.1. Cell Culture: Revival and Expansion of Bone Marrow Mesenchymal Stem Cells (BM-MSC), Adipose Tissue-Derived Mesenchymal Stem Cells (AD-MSC) and Dental Pulp-Derived Mesenchymal Stem Cells (DP-MSC)

Cryopreserved BM-MSC, AD-MSC and DP-MSC (*n* = 5 each) were revived in DMEM-LG medium with 10% FBS (pre-heated to 37 °C). The cells were allowed to adhere to the culture dish by keeping them undisturbed for 24 h and were expanded thereafter. Before cryopreservation, BM-MSCs were obtained by direct plating of bone marrow on to the culture dish and AD-MSC and DP-MSCs were obtained by explant culture. No enzymes were used for the cell extraction.

After expansion of the hMSCs, the cells were characterized by flow cytometric enumeration (Supplementary Materials). Followed by characterization by surface marker profiling using flow cytometry and trilineage differentiation potential of hMSCs, cells from 3rd to 5th passage were used for all further experiments [13]. Briefly, hMSCs were revived in DMEM-LG medium (Gibco, Gaithersburg, MD, USA) with 10% FBS. Upon attaining confluence, cells were characterized by differentiating them into adiopocytes, chondrocytes and osteocytes (trilineage differentiation potential) and were enumerated by flow cytometry for the expression of surface markers like CD105, CD73, CD90, CD29, HLA class 1 & class II and CD34/45 (10,000 cells were acquired for enumeration) [14,15,16]. 

### 2.2. Neuronal Differentiation

The induction medium for neuronal differentiation, contained Neurobasal media (Gibco), B27 supplement (Gibco), FGF2 (50 ng/mL) (PeproTech, Rocky Hill, NJ, USA), forskolin (10 µM), EGF (10 ng/mL), L-Glutamine (Gibco) and PenStrep (Gibco). The induction period was of 14 days with media change on every 3rd day. After completion of the induction period at 14 days, the cells were used for further analytical experiments.

### 2.3. Neurites’ Length Analysis

This was performed as per the already established and published protocol of the lab [13]. Briefly, bright field images of the cells were acquired under microscope and analyzed using SI Viewer software (Tokyo, Japan) for the length and number of neurites, axon length and area and diameter of the cell body. 

### 2.4. Scanning Electron Microscopy (SEM)

Cells for SEM analysis were processed according to the established protocol of the lab [17]. Briefly, hMSCs were cultured and differentiated over cover slips. The cells on coverslips were fixed with Karnovsky fixative (4% paraformaldehyde and 1% glutaraldehyde in 0.1 M Phosphate Buffer (pH 7.4)) for 6–8 h at 4°C and were air-dried later. Dried samples were mounted over aluminium stubs and sputter-coated with gold prior to imaging with EVO18 scanning electron microscope (Zeiss, Oberkochen, Germany) at 5 KVA in secondary electron imaging mode.

### 2.5. Transmission Electron Microscopy (TEM)

Cells for TEM analysis were processed according to the established protocol of the lab [18]. Post-differentiation of hMSCs, medium was removed, and cells were given a gentle wash using PBS (pH 7.4). The cells were then fixed by Karnovsky’s fixative (4% paraformaldehyde and 1% glutaraldehyde in 0.1 M Phosphate Buffer (pH 7.4)) for 6–8 h at 4 °C. Later, gradual dehydration of the cells was performed treating them with a series of ascending concentrations of the ethanol. Finally, ethanol was cleared by treating the cells with xylene. After this, cells were dehydrated in ascending grades of acetone and finally, embedded in araldite CY212. Thin sections (70 nm) were cut with a glass knife and mounted onto nickel grids. They were contrasted with uranyl acetate and lead citrate and viewed under a transmission electron microscope (Tecnai, G 20 (FEI), ThermoScientific, Hillsboro, OR, USA).

### 2.6. Transcriptional Characterization of MSC Induced into Neuronal Cells: Quantitative Reverse Transcription-Polymerase Chain Reaction (qRT-PCR)

After differentiation, total RNA from all the experimental groups was extracted by phenol-chloroform method as previously described [13]. Quantitative analysis of various genes like, *nestin, neurofilament* (*NF*), *β III tubulin* (*Tuj1*), *MAP2, TH*, and transcription factors, *PitX3* and *Ngn2* was performed after 14 days of induction into neuronal cells. Expression of *DAT* (dopamine transporter) gene was also studied. Apart from these, genes related to ion channels like *Kv4.2* (potassium channel) and *SCN5A* (sodium channel) were also studied. Primers of qRT-PCR grade were procured from Sigma (Kalkaska, MI, USA). The expression of the genes of interest was normalized to that of the housekeeping gene, glyceraldehyde-3-phosphate dehydrogenase (*GAPDH*). ΔΔCt method was employed to determine the fold change in the expression of the genes after differentiation as compared to undifferentiated hMSCs. Finally, data were analyzed using the graph pad prism 5.0 software.

### 2.7. Immunocytochemistry

The assay was performed as previously described [13]. Briefly, cells fixed with chilled methanol: acetone and were incubated overnight at 4 °C with primary monoclonal antibodies against MAP2 (1:250), Nestin (1:400), DAT (1:200) and TH (1:200) (Abcam, Cambridge, MI, USA). Treatment with fluoro isothiocyanate (FITC) and texas red (TR) conjugated secondary antibodies (1:500 dil) for 60 min at room temperature. 4′,6-diamidino-2-phenylindole (DAPI) was used to stain and visualize the cell nuclei. Stained cells were examined using a fluorescence microscope equipped with a digital camera (Nikon Eclipse 80i, Tokyo, Japan).

### 2.8. Intracellular Staining for Flow Cytometry

hMSCs after induction were labeled for Nestin (1:100), MAP2 (1:200), TH (1:100), DAT (1:100), synaptophysin (1:100), TPH2 (1:150), GFAP (1:100), S100 (1:100) and Ach (1:150) as described previously [13]. The cells were acquired on BD LSR II flow cytometer (Becton Dickinson, Washington, DC, USA) with minimum of 10,000 events for each sample and analyzed using FACs DIVA software (version 6.1.2). All the antibodies were procured from Abcam.

### 2.9. Immunoblotting

Immunoblotting for the expression of neuronal cell-specific proteins was performed as previously described [13]. Briefly, after preparing whole cell lysates using RIPA buffer (Sigma), BCA Assay method was used for protein quantification. Protein extracts (30 μg) were subjected to SDS-PAGE using 12% Tris/HCl SDS (Sodium dodecyl sulphate) gels and transferred onto PVDF membranes (Membrane Technologies, Ambala, India). 3% BSA at room temperature was used to block the membranes. They were then incubated with primary antibodies against β-actin (Abcam, 1:2500), MAP-2 (Abcam, 1:1500) and TH (Santa Cruz Biotech., Heidelberg, Germany, 1:1000) in 1% BSA-phosphate saline buffer (PBS) at 4 °C overnight. After incubation followed by washing thrice with TBS, membranes were incubated with the appropriate horseradish peroxidase (HRP)-conjugated secondary antibody (1/4000) (Dako, Santa Clara, CA, USA) at room temperature for 2h. Membranes were developed with chemiluminescence detection reagent (Pierce, WA, USA) and acquired by using Gel Imager machine (Fluor Chem E, Cell Biosciences, Preston, VIC, Australia).

### 2.10. Dopamine Release Estimation by Enzyme-Linked Immunosorbent Assay (ELISA)

Amount of dopamine released by induced and a control group was assessed by ELISA using dopamine detection and estimation kit by Elabscience (Houston, TX, USA). The ELISA kit was based on competitive ELISA principle. The assay was performed as per the manufacturer’s protocol. Upon termination of the induction period, the supernatant medium was removed and washed with HBSS. Cells were treated with 54 mM KCl solution and incubated at 37 °C for 5 min. The supernatant was collected and used for DA estimation by the ELISA kit.

### 2.11. Calcium Ion Imaging

Change in the concentration of calcium ions was studied by calcium ion imaging in hMSCs induced for 14 days, as previously described [13]. Briefly, hMSCs were stained with 10 µM of Fura red AM dye for 45 min at 37 °C. After washing thrice with 1X HBSS, the cells were activated using 50 mM KCl solution. Time lapse recording was made at 488 nm and 457 nm for 3 min. Baseline readings were obtained before adding KCl solution to the cells. The experiment was performed using Leica Confocal Microscope (Model TCS SP8). Respective graphs were plotted after obtaining the ratio of florescence at both the wavelengths. The data was analyzed using Leica LAS AF software. 

### 2.12. Data Interpretation and Statistical Analysis

Means ± SD of independent wxperiments were analyzed by Student’s t-test, One Way and Two Way ANOVA test (As per the requirement of data analysis). *p* < 0.05 was considered as statistically significant. Analysis of data was done by using GraphPad Prism 5.00 software (San Diego, CA, USA).

## 3. Results

### 3.1. Dose and Duration Titration of FGF2 and Forskolin to Obtain Desired Neuronal Differentiation Effect Upon Inducing hMSCs 

There are various additives reported with FSK, to differentiate MSCs of various types into neuronal cells. The study by Sujeong et. al. in 2010 [12] reports the induction of human adipose tissue-derived MSCs (AD-MSCs) by using FGF2 (100 ng/mL) for the first seven days, followed by using both FGF2 and FSK (10 µM) for next seven days, of total 14 days of induction period. 

**Figure 1:**
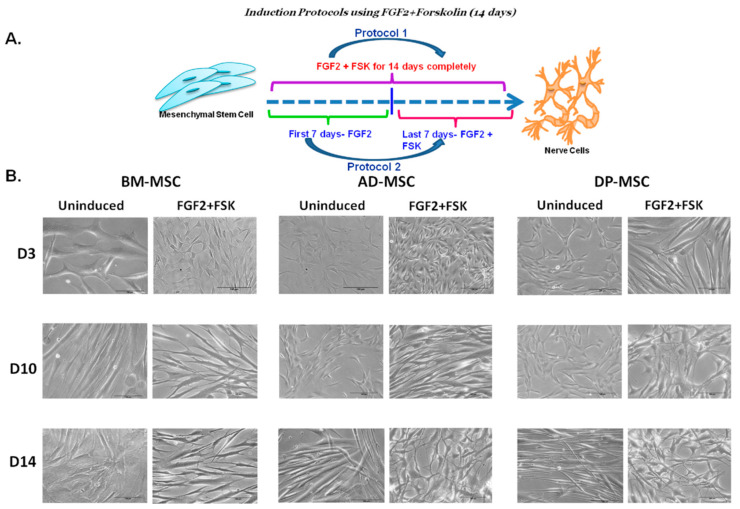
(**A**) Dose and Duration titration of FGF2 and Forskolin to obtain desired neuronal differentiation effect upon inducing human mesenchymal stem cells (hMSCs). (**B**) Change in the cellular morphology of human Mesenchymal Stem Cells was observed upon treatment with FGF2 and forskolin (**A**) (i) BM-MSC; (ii) AD-MSC; (iii) DP-MSC. The morphology of hMSCs has changed from spindle shaped to perikaryl. Appearance of neuronal morphology starts appearing from 6–7th day of induction (scale bar: 100 µ).

Upon performing the dose and duration titration experiment for FSK, it was inferred that only 50 ng/mL of FGF2 is required (as compared to 100 ng/mL reported by other groups) along with FSK (10 µM) to obtain desired neuronal differentiation in hMSCs. This concentration of the combination of FGF2 and FSK is the least reported to date, with similar results in terms of MAP2-positive cells. Hence, it was decided to continue the induction protocol for 14 days with both FGF2 and FSK in the induction cocktail, which was lower than that reported earlier by other groups (Figure 1A).

### 3.2. Change in the Cellular Morphology of Human Mesenchymal Stem Cells Was Observed upon Treatment with FGF2 and Forskolin

**Figure 2:**
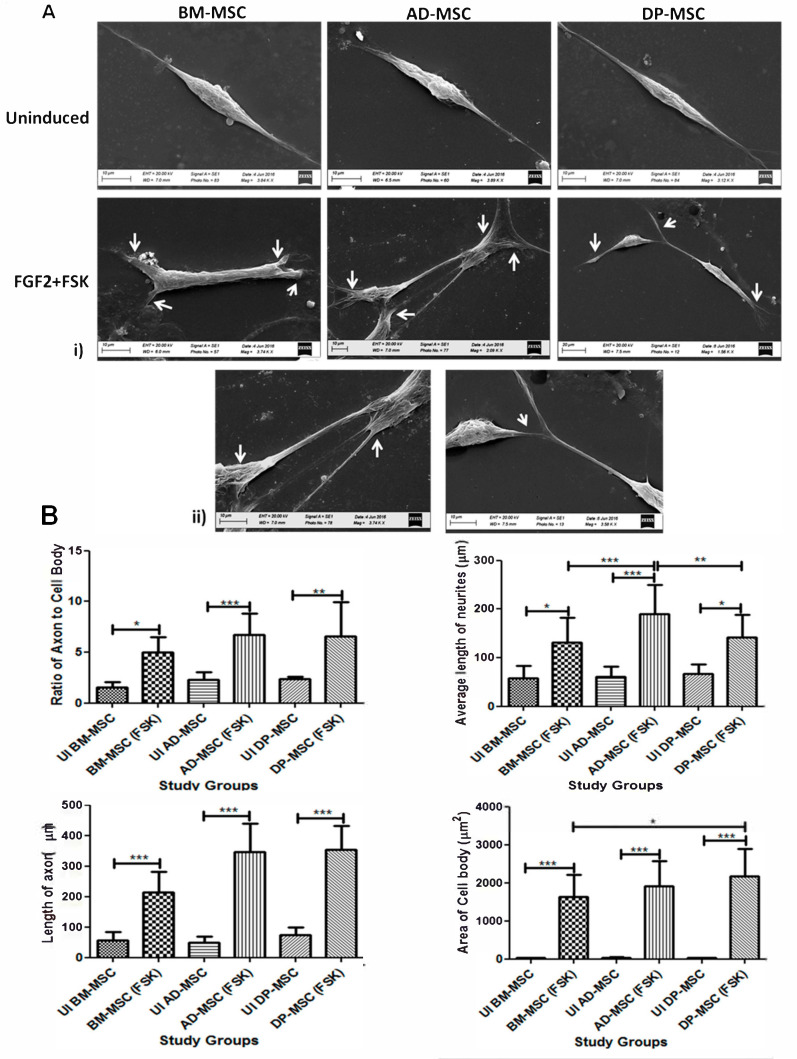
(**A**) Scanning Electron Microscopic observations depicting minute morphological changes occurring in hMSCs after neuronal induction (**i**) Morphology of hMSCs has changed from spindle shaped to perikaryl. Terminals of the cells show appearance of minute neurites-like structures, (**ii**) Magnified images of differentiated cells, showing the appearance of axon-hillock, neuritogenesis, and appearance of terminal neurites, facilitating interactions. (**B**) Forskolin leads to increase in the neurites’ length, axonal development, appearance of distinct nucleus and marked change in the morphology of human Mesenchymal stem cells. For studying all the parameters under the study, five different samples of each type of hMSC were taken (*n* = 40 for each study group). Data was analyzed by three independent observers (*p* < 0.05) (*, ** and *** represent the decimal points of significance).

Upon induction of hMSCs with FGF2 and FSK, basic morphological changes were observed. The typical spindle-shaped morphology of hMSCs started changing to the appearance of distinct and bulbous cell body and cytoplasmic extensions. FSK induced neurite lengthening or branching in hMSCs obtained from all tissue sources. Nucleus of the cell shifted towards the periphery and developed cytoplasmic extensions, through axon hillock-like structures on the cell body. The terminals of the induced cells were also observed to have multiple dendritic structures. Cells after differentiation showed profound nucleus, more neurites and interconnections between different cells in the culture (Figure 1B). These preliminary features were confirmed by scanning electron microscopic (SEM) studies, showing development of fine neurites at the terminals of hMSCs post-differentiation. Several fields also showed the cell to cell interaction, with extended neurites like extensions. Axon-hillock like structures were observed in the differentiated hMSCs (Figure 2A).

However, uninduced hMSCs were more flattened with spindle shape and had a centralized nucleus. 

### 3.3. Forskolin Leads to Increase in the Neurites’ Length, Axonal Development, Appearance of Distinct Nucleus and Marked Change in the Morphology of Human Mesenchymal Stem Cells 

Neuritogenesis and axonogenesis in the induced cells were assessed in detail by measuring the average area of the cell body. Average of the cell body increased from 35.3 ± 0.8 µm^2^ to 1578 ± 110.3 µm^2^ in BM-MSCs, from 41.06 ± 2.9 µm^2^ to 1891 ± 127.5 µm^2^ in AD-MSCs and from 34.65 ± 1.7 µm^2^ to 2112 ± 148.9 µm^2^ in DP-MSCs. Average length of the neurites increased from 58.54 ± 12.32 µm to 135.2 ± 11.26 µm in BM-MSCs, from 51.79 ± 6.8 µm to 190.2 ± 10.28 µm in AD-MSCs and from 65.36 ± 11.0 µm to 142.06 ± 9.4 µm in DP-MSCs. When axon length (longest neurite) was calculated, an upsurge from 58.69 ± 11.22 µm to 217.4 ± 14.53 µm in BM-MSCs, from 50.02 ± 7.05 µm to 351.1 ± 16.88 µm in AD-MSCs and from 78.16 ± 10.19 µm to 342.8 ± 15.4 µm in DP-MSCs was observed. There was significant difference observed in the area of cell body between AD-MSC and DP-MSC only (Figure 2B).

### 3.4. Treatment of Human Mesenchymal Stem Cells with FGF2 and Forskolin Is Marked by an Increased Expression of Neuronal and DAergic Neuronal Genes

**Figure 3:**
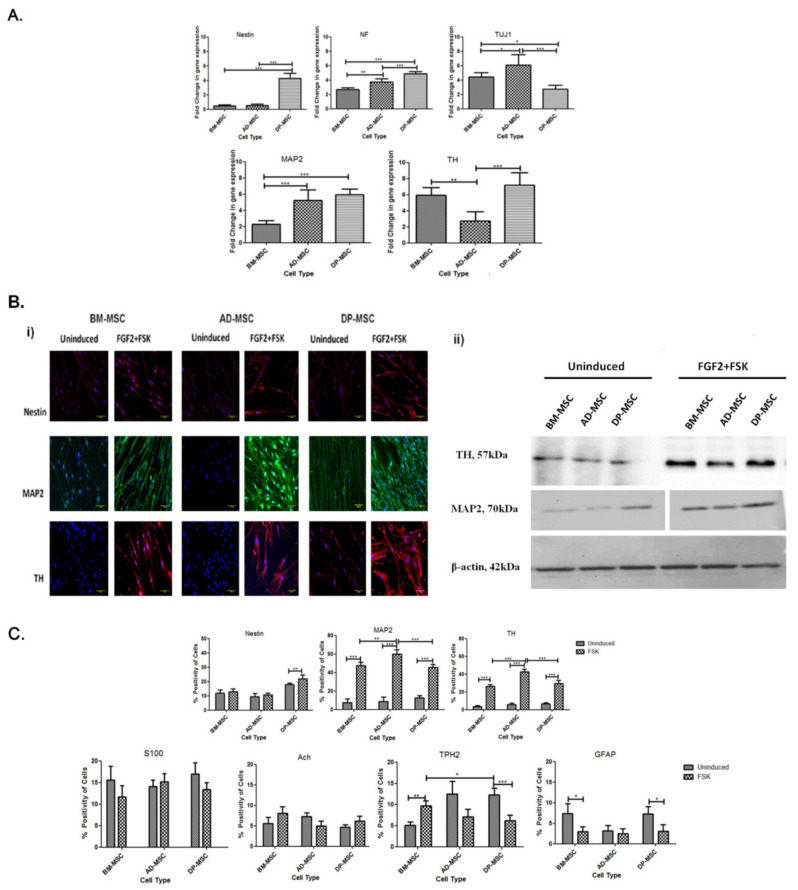
(**A**) Treatment of human Mesenchymal stem cells with FGF2 and forskolin is marked by an increased expression of neuronal and dopaminergic (DAergic) neuronal genes. Graphs depicting the relative fold change in mRNA expression *of nestin, neurofilament (NF), β III tubulin (TUJ1), MAP2,* and *tyrosine hydroxylase (TH)* in induced hMSCs, as compared to respective uninduced hMSCs (*, *p* < 0.05) (**B**) An upsurge in the expression of neuronal proteins followed the upregulation of neuronal genes: (**i**) Immunoflorescence Assay showing expression of nestin, MAP2, and TH protein expression in hMSCs pre- and post-differentiation into DAergic neuronal cells; (**ii**) Immunoblotting Assay for expression of neuronal and DA neuronal cell-associated proteins (MAP2 and TH) in uninduced and differentiated hMSCs. (**C**) Flow cytometric enumeration reveals an increase in the percentage of cells positive for neuronal and dopaminergic neuronal proteins post-differentiation of hMSCs: (i) Graphs depicting the number of nestin, MAP2- and TH-positive cells pre- and post-neuronal induction. DP-MSC have maximum number of nestin-positive (*, *p* < 0.05), cells at the beginning of experiments, followed by those in AD-MSC and BM-MSC. (ii) Infinitesimal percentage of cells positive for non-dopaminergic markers were observed post-induction (*, *p* < 0.05) (*, ** and *** represent the decimal points of significance).

Induced hMSCs were characterized for the expression of neuronal and DAergic neuronal-specific genes. After induction a relative upregulation was observed in the expression of neurofilament (NF) (2.7 ± 0.11, 3.7 ± 0.2 and 4.9 ± 0.14 folds in BM-MSCs, AD-MSCs and DP-MSCs, respectively), Neuron-specific class III beta-tubulin (TUJ1) (4.4 ± 0.28, 6.1 ± 0.64 and 2.7 ± 0.25 folds in BM-MSCs, AD-MSCs and DP-MSCs, respectively), Microtubule-associated protein 2 (MAP2) (2.2 ± 0.2, 5.2 ± 0.6 and 5.9 ± 0.32 folds in BM-MSCs, AD-MSCs and DP-MSCs, respectively) and Tyrosine Hydroxylase (TH) (5.9 ± 0.42, 2.7 ± 0.5 and 7.2 ± 0.7 folds in BM-MSCs, AD-MSCs and DP-MSCs, respectively). However, there was no significant increase observed in transcriptional expression of nestin, except that in DP-MSCs with 4.3 ± 0.4 folds (Figure 3A).

No specific trend in the expression of various genes was observed in this case, except in the case of DP-MSC, which showed maximum upregulation of the above-mentioned genes. However, AD-MSCs showed comparable expression of genes and proteins with that in DP-MSCs. 

### 3.5. An Upsurge in the Expression of Proteins Followed the Upregulation of Neuronal and DAergic Neuronal Genes 

Upon termination of induction period of 14 days, differentiated hMSCs were labelled for neuronal cell-specific proteins nestin, MAP-2 and DAergic sub-specification protein, TH. Induced cells were found to be positive for these markers as compared to uninduced groups (Figure 3Bi). There was a minimal basal expression of MAP2 & TH in uninduced hMSCs, while an upregulated expression was observed post-induction in all the hMSC types. An increased fluorescence intensity (as compared to that in respective uninduced hMSCs) in the images support higher expression of MAP2 and TH in differentiated cells. However, among the various hMSCs types, highest fluorescence intensity of these protein markers was observed in case of DP-MSC. Similar trend was also observed when immunoblotting assay was performed in both uninduced and induced groups (Figure 3Bii). 

### 3.6. Flow Cytometric Enumeration Reveals an Increase in the Percentage of Cells Positive for Neuronal and Dopaminergic Neuronal Proteins Post-Differentiation hMSCs

hMSCs were enumerated for neuronal and DAergic neuronal cells related proteins by flow cytometry. Except in the case of DP-MSCs (from 17.84 ± 0.46% to 21.88 ± 1.26%), no significant increase in the percentage of cells positive for nestin (from 11.84 ± 1.08% to 13.01 ± 0.86% in BM-MSCs and from 9.460 ± 0.96% to 10.78 ± 0.45% in AD-MSCs) was observed. This observation may be attributed to the difference in the mode of action of FGF2 and FSK on different hMSCs under study. Another possible reason could be the propensity of DP-MSCs towards neural lineage, due to their origin from neural crest. MAP2-positive cells were significantly increased post-induction in all hMSC types, under the study. However, maximum upsurge was observed in AD-MSCs (59.80 ± 2.136%), followed by that in BM-MSCs (47.54 ± 1.5%) and DP-MSCs (45.28 ± 1.6%). The difference in the outcomes of BM-MSCs and DP-MSCs was non-significant; unlike that between AD-MSCs and rest of the two hMSC types. These observations of flow cytometric analysis state that FGF2 and FSK led to an increase in percentage of MAP2-positive cells higher in AD-MSCs only as compared to BM-MSCs and DP-MSCs (Figure 3Ci). 

TH-positive cells were enumerated to study about the DAergic specification of sells post-differentiation. It showed a similar trend as MAP2-positive cells. While no significant difference was observed in the percentage of cells positive for TH between BM-MSCs and DP-MSCs post-neuronal induction (26.20 ± 0.8390% and 29.64 ± 1.461%, respectively), 42.78 ± 1.248% cells were found to be positive for TH in AD-MSCs. To the best of our knowledge, no study has been reported to date to comment on the percentage efficiency of generation of DAergic neuronal cells using this protocol (Figure 3Ci).

### 3.7. Infinitesimal Percentage of Cells Positive for Non-Dopaminergic Markers Were Observed Post-Induction

Cell milieu of the induced hMSCs consisted of cell positive for non-DAergic neuronal proteins like Ach (acetylcholinergic neurons), TPH2 (serotonergic neurons), S100 (Schwann cells) and GFAP (glial cells). Upon flow cytometric enumeration, it was observed that the percentage of TPH2-positive cells has increased significantly only in BM-MSC (from 5.04 ± 0.37% to 9.62 ± 0.5580%); while it decreased significantly in AD-MSCs (from 12.46 ± 1.349% to 7.040 ± 0.81%) and DP-MSCs (from 12.28 ± 0.73% to 6.16 ± 0.57%). Upon enumeration of Ach-positive cells in the culture after induction, a non-significant increase was observed in cases of BM-MSCs and DP-MSCs. However, decrease in the cells positive for Ach was observed with AD-MSCs. However, cells positive for S100 were only increased in AD-MSCs post-induction, however, non-significantly. There was a significant decrease in GFAP-positive cells in BM-MSCs and AD-MSCs, after induction; while AD-MSCs showed no significant difference (Figure 3Cii). 

### 3.8. Increase in the Genes and Proteins Expression, Responsible for Functionality of Neuronal and Dopaminergic Neuronal Cells

#### 3.8.1. mRNA Expression of Transcription Factors Responsible for Survival and Maintenance of DAergic Neuronal Cells 

**Figure 4:**
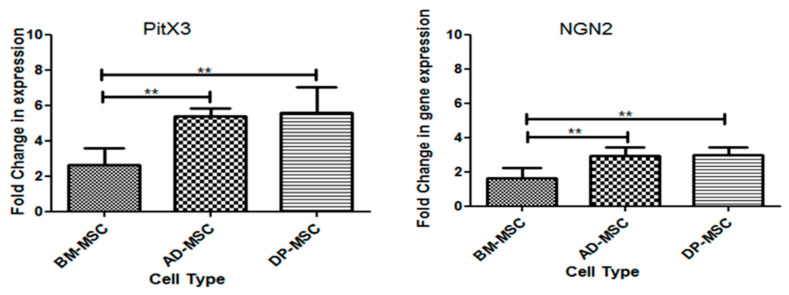
mRNA expression of transcription factors responsible for survival and maintenance of DAergic neuronal cells (*p* < 0.05) (** represent the decimal points of significance).

FSK also increased the expression of transcription factors (TFs), which are responsible for the maturation and survival of DAergic neurons [19]. The relative fold change in the expression level of Neurogenin 2 (NGN2) and paired-like homeodomain transcription factor 3 or pituitary homeobox 3 (PitX3) was investigated in induced hMSCs, as compared to their uninduced controls. It was observed that PitX3 and NGN2 followed similar trend in their expression with the three types of hMSCs under the study. Both PitX3 and ngn2 were maximally upregulated in AD-MSCs (5.4 ± 0.19 folds change in PitX3 and 2.93 ± 0.21 folds change in ngn2) and DP-MSCs (5.58 ± 0.65 folds change in PitX3 and 2.970 ± 0.1984 folds change in ngn2), with no significant difference, except in BM-MSCs (2.66 ± 0.42 folds change in PitX3 and 1.63 ± 0.27 folds change in ngn2), showing significant difference. These results support that the differentiated hMSCs expressed prototypical midbrain DAergic markers at mRNA level, with difference in the expression level, within themselves (Figure 4).

#### 3.8.2. FSK Improves Functional Dopaminergic Specifications at Both Gene and Protein Level

**Figure 5:**
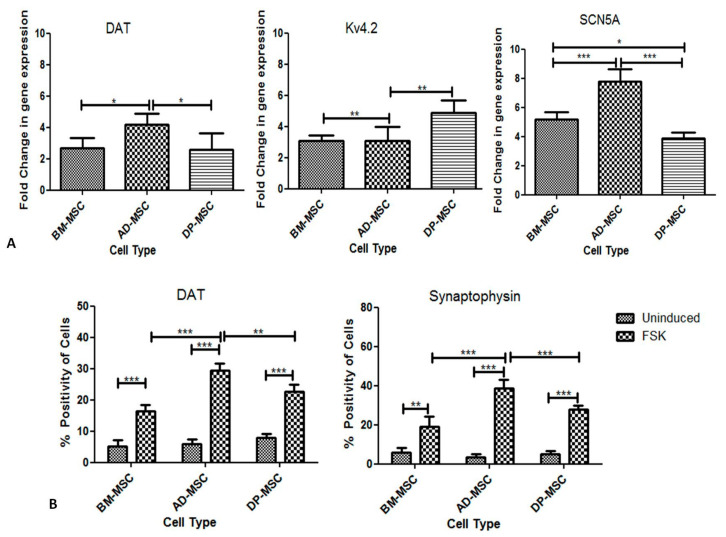
Forskolin (FSK) improves functional dopaminergic specifications at both gene and protein level: (**A**) qRT-PCR mRNA transcriptional analysis of differentiated hMSCs for genes associated with functionality of DAergic neuronal cells (*p* < 0.05). (**B**) Flow cytometric analysis for (i) Dopamine transporter protein, responsible for releasing dopamine neurotransmitter through vesicles (*p* < 0.05) and (ii) Synaptophysin protein, which is responsible for synapse formation between two neuronal cells (*p* < 0.05) (*, ** and *** represent the decimal points of significance).

Characterization of differentiated hMSCs for their functionality was performed at gene level by studying the relative fold change in the mRNA expression level of *Kv4.2*, *SCN5A* and *DAT*. *DAT* showed highest upregulation in AD-MSCs (4.16 ± 0.32 change folds), followed by that in BM-MSCs (2.66 ± 0.31 change folds) and DP-MSCs (2.56 ± 0.46 change folds), with no significant difference. Likewise, *SCN5A* had highest upregulation in AD-MSCs (7.8 ± 0.36 change folds), followed by that in BM-MSCs (5.2 ± 0.21 change folds) and DP-MSCs (3.8 ± 0.19 change folds), with no significant difference. However, folds change in the expression of *Kv4.2* was observed to be highest in DP-MSCs (4.9 ± 0.36 change folds) followed by BM-MSCs (3.0 ± 0.14 change folds) and AD-MSCs (3.09 ± 0.38 change folds) with no significant difference. Degree of upregulation of functionality related genes was significantly higher in differentiated AD-MSCs, as compared to that in other hMSCs, except for *Kv4.2* (Figure 5A).

DAT- and synaptophysin-positive cells were enumerated by flow cytometry. It was discerned that cells positive for both these proteins were significantly higher in differentiated hMSC as compared to that in uninduced ones. Differentiated AD-MSCs had maximum percentage of cells positive for DAT (29.48 ± 0.99%) as compared to that in DP-MSCs (22.78 ± 0.90%) and BM-MSCs (16.36 ± 0.93%). 

Furthermore, highest number of synaptophysin-positive cells were found in differentiated AD-MSCs (38.56 ± 2.07%), followed by that in DP-MSCs (28.02 ± 0.77%) and BM-MSCs (19.20 ± 2.29%). This difference was significant amongst all the types of induced hMSCs (Figure 5B). 

#### 3.8.3. Neuronal Differentiation Led to Changes in the Cells at Ultra-Structural Level 

Neuronal differentiation of hMSCs is linked with the ultra-structural modifications in the mitochondria [20], dense core vesicles or granules (DCVs) [21], rough endoplasmic reticulum (RER) [22], cytoplasmic filamentous condensation [23] and endocytotic vesicles [24]. 

According to our results, higher numbers of mitochondria were observed in differentiated hMSCs, showing increased mitochondrial biogenesis. These mitochondrial were observed to have globular cup-like structure and evident cristae. 

Upon differentiation of hMSCs, an increase in the number of DCVs (these are specialized secretory organelles which are rich in ADP, ATP, histamine, ionized calcium, and neurotransmitters) and RER (help in axonogenesis and dendritogenesis), contributing in keeping the neuronal cells functional. The increase in the number of DCVs and RER may be linked to increased functionality of the cells. More DCVs may also be associated with the chemical functionality of the DAergic neurons, required for the secretion of neurotransmitters.

**Figure 6:**
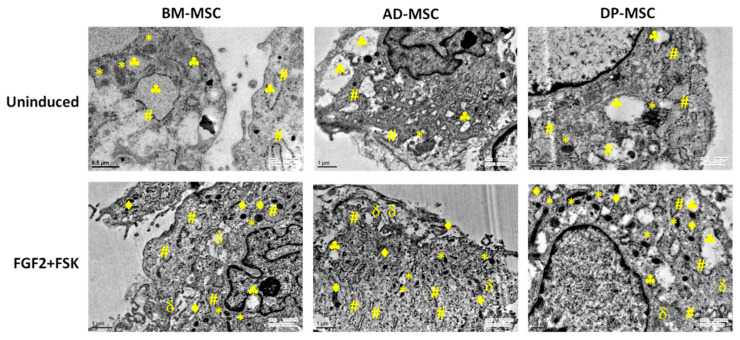
Neuronal differentiation led to changes in the cells at ultra-structural level: Ultra-structural composition of hMSCs has changed on various parameters. There was observed increased mitogenesis, increase in dense core vesicles, rough endoplasmic reticulum, cytoskeletal condensation, and endocytotic vesicles. The genesis of these cellular organelles may be associated with the increased functionality of the terminally differentiated hMSCs. Here, # represent rough endoplasmic reticulum, * represent mitochondria, ♣ represent endocytotic vacuoles, δ represent cytoskeletal condensation and ♦ represent dense core vesicles.

Cytoskeletal rearrangement is essential for maintaining cellular morphology, regulates growth cone motility, forms scaffold for transport of mitochondria and other organelles and for axon guidance. Microtubules were observed to be arranged in a synchronized pattern after differentiation of hMSCs with FGF2 and FSK, which is an indicator of neuritogenesis and axonogenesis (Figure 6).

### 3.9. Induced Human Mesenchymal Stem Cells Release Higher Concentration of Dopamine in the Medium

**Figure 7:**
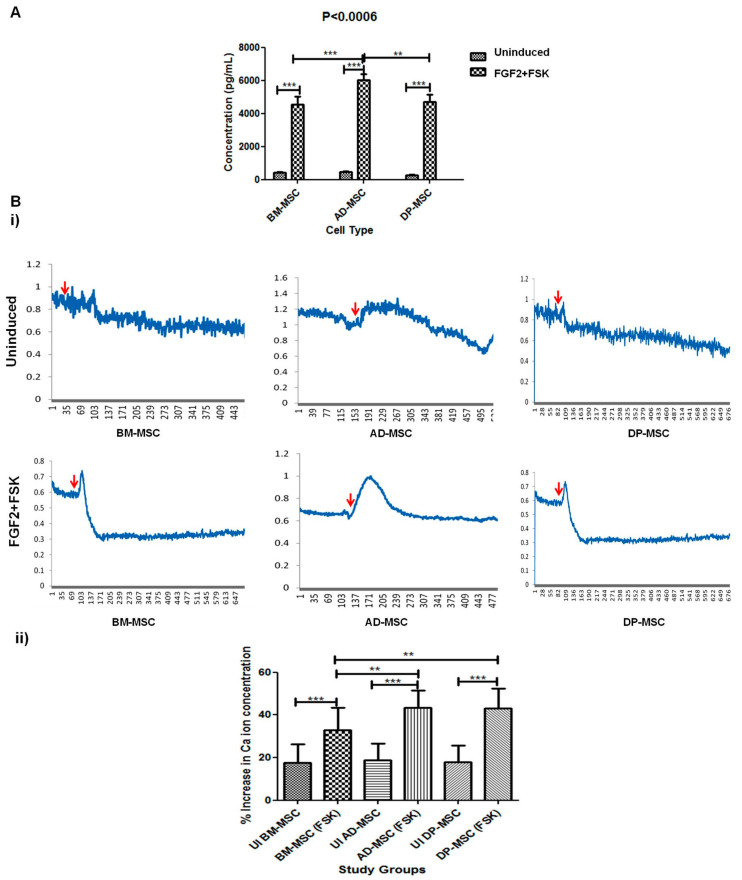
(**A**) Induced human Mesenchymal Stem Cells release higher concentration of dopamine in the medium as depicted by ELISA (*p* < 0.05) (**B**) Significantly higher change in the calcium ion efflux was observed in human Mesenchymal stem cells post-induction (**i**) Graphs showing the changes in the Ca^2+^ transients upon depolarization with KCl in cells of all cell types. The uninduced hMSC did not show any change in the intracellular Ca^2+^ concentration after depolarization. Red arrows indicate the time point of addition of KCl in the culture medium for depolarization; (**ii**) Graph showing change in the percentage increase in the calcium ion concentration in various hMSCs after DAergic neuronal induction by FGF2 and FSK (*p* < 0.05) (** and *** represent the decimal points of significance).

Chemical functionality of induced hMSCs is defined by the amount of dopamine released by them in the medium. When cells were evoked with KCl solution, dopamine was released in the solution. A baseline amount of dopamine was observed in case of all three types of hMSCs under study. However, induced cells released significantly higher amount of dopamine in the solution, highest being released by induced AD-MSCs (6047 ± 210.9 pg/mL), followed by DP-MSCs (4716 ± 245.3 pg/mL) and BM-MSCs (4562 ± 266.3 pg/mL) (Figure 7).

### 3.10. Significantly Higher Change in the Calcium Ion Efflux Was Observed in Human Mesenchymal Stem Cells Post-Induction

The change in the calcium ion concentration in the cytosol of the in vitro generated neuronal cells was observed, after adding 50 mM KCl solution. A significant difference in the intracellular calcium ion transients was noted in DAergic neuronal cells, generated by treatment with FGF2 and FSK in all hMSCs types, as compared to that in respective uninduced MSCs. In AD-MSCs the change in calcium ion transients was observed to be maximum (43.31 ± 1.77%) as compared to the uninduced group (18.75 ± 1.71%). This was followed by that in DP-MSCs (43.11 ± 1.87% in differentiated and 17.93 ± 1.73% in control group) with no significant difference, and least calcium ion transients were observed in case of induced BM-MSCs (33.00 ± 2.26% in differentiated and 17.57 ± 1.91% in control group). The change in the calcium ion transients in induced BM-MSCs was significantly lower than that observed in AD-MSCs and DP-MSCs. The change in the calcium ion concentration in various cell types has been detailed in Figure 7.

## 4. Discussion

Stem cells have come up as promising candidates for treating several neurodegenerative diseases like Parkinson’s disease, Alzheimer’s disease, and motor neuron disease. Mesenchymal stem cells are the most reliable cell candidates for both autologous and allogenic stem cell transplantation to treat such types of neuro-degenerative diseases. These MSCs can be harvested from a number of tissue sources like bone marrow, adipose tissue, dental pulp, Wharton’s jelly, and amniotic fluid [25,26,27]. The neuro-regenerative potential of MSCs obtained from all these tissue sources have been explored widely by several research groups. The upcoming interest of researchers and clinicians is to transplant neuronal cells primed from these stem cells for better prognosis of the disease. Hence, the current research focus in this field is to devise the optimum differentiation protocol, which is cost effective, efficient, and targeted towards generation of functional neuronal cells. This will not only help in translational medicine, but also in drug testing and understanding neurogenesis at the basic research level. Hence, the current study was based on exploring the neurogenic potential of forskolin, a cAMP activator. 

There are very few studies reporting the use of FSK for in vitro neurogenesis of MSCs [10,15,28]. Most of these studies have presented the neurogenic use of FSK along with a number of other chemicals and growth factors like FGF2, EGF, DHA, AMP, IBMX, RA, and NGF. However, these chemical inducers cannot be used to differentiate hMSCs for clinical use as they have been known to cause changes in the cell cycle. Moreover, their other side effects have also not been explored widely. As forskolin is a natural resin and already has several medicinal uses, it may be used further for clinical purpose, in terms of differentiation of stem cells. To the best of our knowledge, to date, there are only few studies reporting the use of FSK along with FGF2 only, with the induction period of 14 days, for differentiating AD-MSCs into neuronal cells [29,30,31]. These studies state the characterization of differentiated cells on the basis of various markers like nestin, TUJ1, MAP2, NF-L, NF-M, NF-H, NSE, NeuN, GAP43, and functionality assessment by the gene expression of channel ion related proteins and synaptic markers like SNAP25. The group has also studied GFAP- and CNPase-positive cells in the culture after induction by immunoflorescence. Membrane depolarization studies were also performed and reported. Another recent study reports human umbilical cord-derived MSCs as the cell candidate to perform in vitro differentiation studies with forskolin and IBMX as neuronal inducers [30]. However, these studies do not report an efficient protocol of generation of functional dopaminergic neuronal cells. Also, there is no comparative study reported using FGF2 and forskolin together as neuronal inducer for hMSCs obtained from various tissue sources.

In the current study, we have performed detailed morphological, morphometric and ultra-structural studies of the in vitro generated DAergic neuronal cells from hMSCs. Then we have characterized the DAergic neuronal cells at gene and protein levels. We have obtained a good efficiency of 59.80 ± 2.136% of generation of mature neuronal cells and 42.78 ± 1.248% DAergic neuronal cells with AD-MSCs as the stem cell candidates, as is evident by number of MAP2- and TH-positive cells, respectively, post-differentiation. The number of cells positive for DAT and synaptophysin were also higher in differentiated AD-MSCs. No significantly higher difference was observed with DP-MSCs, in spite of showing more nestin-positive cells. We emphasize more on AD-MSCs in our paper, as they have higher translational implication. This data is comparable with already reported results with other MSC types [13,30,31,32,33,34,35]. We have also performed the studies and commented on the changes occurring at the sub-cellular levels in the MSCs after differentiation into DAergic neuronal cells. Our study also reports the functional assessment of the in vitro generated DAergic neuronal cells at gene and protein levels. Dopamine released by the induced cells was also significantly higher in AD-MSCs, as compared to other cell types. This observation is in line with all our other experiments. However, the amount of dopamine release by using FGF2 and FSK has not been reported till now. This concentration of dopamine release is higher than the previously reported studies using other protocols [30,31,32,34]. Calcium ion imaging was performed to assess the membrane depolarization of the cells upon treatment with 50 mM KCl solution. All of these studies have shown that FGF2 and FSK have maximum neurogenic effect on AD-MSCs. BM-MSCs showed upregulation of markers associated with mature neuronal and DAergic neuronal cells, but that was significantly lower than that observed with AD-MSCs. DP-MSCs showed good outcomes in some cases, which were comparable to those obtained in case of AD-MSCs.

Since, a difference in the neurogenic effect of FGF2 and FSK has been observed with the three types of hMSCs under the study, it may be attributed to the difference in the pathways being activated with FGF2 and FSK. Origin of these tissues at embryonic level should also be considered while devising any pathways study to unravel the functioning of FGF2 and FSK. Wnts are the family of glycoproteins that can signal through various intracellular pathways and thus, help in regulation of several developmental events in mammals [36,37]. Wnt proteins show their effect through frizzled receptors, which are seven membrane proteins and are considered to be the first receptors to transducer Wnt signals [38]. Wnt/Fzd complex sends signals by the activation of Dishevelled (Dvl) protein [39] in the cytoplasm to inhibit the degradation of β-catenin by the glycogen synthase kinase-3β (GSK3β). GSK3β is required to induce transcription of target gene expression. The Wnt signaling pathway plays a vital role in the development and functioning of central nervous system. It also plays an imperative role in neuronal differentiation [40]. Specifically, Wnt3a and Wnt5a have been reported to play most crucial role in neuronal differentiation by increasing the deposition of β-catenin, in case of rat MSCs [41]. 

To date, very few studies only have been reported to comment upon the role of Wnt signaling or β-catenin signaling in neural-induced human AD-MSCs [25,41,42,43]. Our study holds the basis for future research in terms of exploration of pathways and biomolecules activated by FGF2 and FSK during the course of neurogenesis. To the best of our knowledge, this is the first study, which reports the in-depth characterization of in vitro generated DAergic neuronal cells at morphological, transcriptional, translational, and functional levels. However, further pathway studies are required to unravel the possible reasons for differential behavior of various types of BM-MSCs, AD-MSCs and DP-MSCs with this induction protocol.

## 5. Conclusions

From the study conducted and data analyzed, it may be concluded that a combination of FGF2 and forskolin yields functional dopaminergic neuronal cells from hMSCs obtained from bone marrow, adipose tissue, and dental pulp. However, the most efficient hMSC for this induction protocol was found to be AD-MSC. However, in vivo investigations are required to establish the translational implications of the protocol and hMSCs.

## Supplementary Materials

The following are available online at https://www.mdpi.com/2073-4409/9/9/2058/s1, Figure S1: Isolation, expansion, characterization of hMSCs. Figure S2: Proliferation assay of hMSCs.

## References

[1] Seamon K.B., Padgett W., Daly J.W. (1981). Forskolin: Unique diterpene activator of adenylate cyclase in membranes and in intact cells. Proc. Natl. Acad. Sci. USA.

[2] Liu M.L., Zang T., Zou Y., Chang J.C., Gibson J.R., Huber K.M., Zhang C.L. (2013). Small molecules enable neurogenin 2 to efficiently convert human fibroblasts into cholinergic neurons. Nat. Commun..

[3] Fimia G.M., Sassone-Corsi P. (2001). Cyclic AMP signalling. J. Cell Sci..

[4] Cheng X., Ji Z., Tsalkova T., Mei F. (2008). Epac and PKA: A tale of two intracellular cAMP receptors. Acta Biochim. Biophys. Sin..

[5] Taran R., Mamidi M.K., Singh G., Dutta S., Parhar I.S., John J.P., Bhonde R., Pal R., Das A.K. (2014). In vitro and in vivo neurogenic potential of mesenchymal stem cells isolated from different sources. J. Biosci..

[6] Pittenger M.F., Discher D.E., Péault B.M., Phinney D.G., Hare J.M., Caplan A.I. (2019). Mesenchymal stem cell perspective: Cell biology to clinical progress. NPJ Regen. Med..

[7] Rendra E., Scaccia E., Bieback K. (2020). Recent advances in understanding mesenchymal stromal cells. F1000Research.

[8] Elgaz S., Kuçi Z., Kuçi S., Bönig H., Bader P. (2019). Clinical Use of Mesenchymal Stromal Cells in the Treatment of Acute Graft-versus-Host Disease. Transfus. Med. Hemother..

[9] Levy Y.S., Bahat-Stroomza M., Barzilay R., Burshtein A., Bulvik S., Barhum Y., Panet H., Melamed E., Offen D. (2008). Regenerative effect of neural-induced human mesenchymal stromal cells in rat models of Parkinson’s disease. Cytotherapy.

[10] Rooney G.E., Howard L., O’Brien T., Windebank A.J., Barry F.P. (2009). Elevation of cAMP in mesenchymal stem cells transiently upregulates neural markers rather than inducing neural differentiation. Stem Cells Dev..

[11] Friedenstein A.J., Piatetzky S., Petrakova K.V. (1966). Osteogenesis in transplants of bone marrow cells. J. Embryol. Exp. Morphol..

[12] Friedenstein A.J., Petrakova K.V., Kurolesova A.I., Frolova G.P. (1968). Heterotopic of bone marrow: Analysis of precursor cells for osteogenic and hematopoietic tissues. Transplantation.

[13] Friedenstein A.J., Chailakhjan R.K., Lalykina K.S. (1970). The development of fibroblast colonies in monolayer cultures of guinea-pig bone marrow and spleen cells. Cell Tissue Kinet..

[14] Hedvika D., Xiufang G., Stephen L., Maria S., James J.H. (2011). Small molecule induction of human umbilical stem cells into myelin basic protein positive oligodendrocytes in a defined three-dimensional environment. ACS Chem. Neurosci..

[15] Sujeong J., Hyong-Ho C., Yong-Bum C., Jong-Seong P., Han- Seong J. (2010). Functional neural differentiation of human adipose tissue-derived stem cells using bFGF and forskolin. BMC Cell Biol..

[16] Singh M., Kakkar A., Sharma R., Kharbanda O.P., Monga N., Kumar M., Chowdhary S., Airan B., Mohanty S. (2017). Synergistic Effect of BDNF and FGF2 in Efficient Generation of Functional Dopaminergic Neurons from human Mesenchymal Stem Cells. Sci. Rep..

[17] Jain K.G., Mohanty S., Ray A.R., Malhotra R., Airan B. (2015). Culture & differentiation of mesenchymal stem cell into osteoblast on degradable biomedical composite scaffold: In vitro study. Indian J. Med. Res..

[18] Sen S., Sharma S., Gupta A., Gupta N., Singh H., Roychoudhury A., Mohanty S., Sen S., Nag T.C., Tandon R. (2011). Molecular characterization of explant cultured human oral mucosal epithelial cells. IOVS.

[19] Sunghoi H., Sangmi C., Kaka L., Insik H., Jisook M., Kwang-Soo K. (2013). Functional Roles of Nurr1, Pitx3, and Lmx1a in Neurogenesis and Phenotype Specification of Dopamine Neurons During In Vitro Differentiation of Embryonic Stem Cells. Stem Cells Dev..

[20] Agostini M., Romeo F., Inoue S., Niklison-Chirou M.V., Elia A.J., Dinsdale D., Morone N., Knight R.A., Mak T.W., Melino G. (2016). Metabolic reprogramming during neuronal differentiation. Cell Death Differ..

[21] Malosio M.L., Giordano T., Laslop A., Meldolesi J. (2004). Dense-core granules: A specific hallmark of the neuronal/neurosecretory cell phenotype. J. Cell Sci..

[22] Yumei W., Christina W.C., Shan X., Hayworth J., Kenneth W., Richard J., Hess F., Harald F., Camilli P.D. (2017). Contacts between the endoplasmic reticulum and other membranes in neurons. Proc. Natl. Acad. Sci. USA.

[23] Compagnucci C., Piemonte F., Sferra A., Piermarini E., Bertini E. (2016). The cytoskeletal arrangements necessary to neurogenesis. Oncotarget.

[24] Patton R.G., Simons K., Dotti C.G. (1992). Axonal and Dendritic Endocytic Pathways in Cultured Neurons. J. Cell Biol..

[25] Hass R., Kasper C., Böhm S., Jacobs R. (2011). Different populations and sources of human mesenchymal stem cells (MSC): A comparison of adult and neonatal tissue-derived MSC. Cell Commun. Signal. CCS.

[26] Giai Via A., Frizziero A., Oliva F. (2012). Biological properties of mesenchymal Stem Cells from different sources. Musclesligaments Tendons J..

[27] Ledesma-Martínez E., Mendoza-Núñez V.M., Santiago-Osorio E. (2016). Mesenchymal Stem Cells Derived from Dental Pulp: A Review. Stem Cells Int..

[28] Paldino E., Cenciarelli C., Giampaolo A., Milazzo L., Pescatori M., Hassan H.J., Casalbore P. (2014). Induction of dopaminergic neurons from human Wharton’s jelly mesenchymal stem cell by forskolin. J. Cell. Physiol..

[29] Shahbazi A., Safa M., Alikaram F., Kargozar S., Asadi H.M., Joghataei T., Soleimani M. (2016). Rapid Induction of Neural Differentiation in Human Umbilical Cord Matrix Mesenchymal Stem Cells by cAMP-elevating Agents. Int. J. Mol. Cell Med. Summer.

[30] Tio M., Kia H.W., Wendy L., Wang T.T., Udolph G. (2010). Roles of db-cAMP, IBMX and RA in aspects of neural differentiation of cord blood derived mesenchymal like stem cells. PLoS ONE.

[31] Trzaska K.A., King C.C., Li K.Y., Kuzhikandathil E.V., Nowycky M.C., Ye J.H., Rameshwar P. (2009). Brain-derived neurotrophic factor facilitates maturation of mesenchymal stem cell-derived dopamine progenitors to functional neurons. J. Neurochem..

[32] Trzaska K.A., Kuzhikandathil E.V., Rameshwar P. (2007). Specification of a dopaminergic phenotype from adult human mesenchymal stem cells. Stem Cells.

[33] Thompson R., Casali C., Chan C. (2019). Forskolin and IBMX Induce Neural Transdifferentiation of MSCs Through Down regulation of the NRSF. Sci. Rep..

[34] Sachetti P., Sousa K.M., Hall A.C., Liste I., Steffensen K.R., Theofilopoulos S., Parish C.L., Hazenberg C., Richter L.A., Hovatt O.A. (2009). Liver X Receptors and oxysterols promote ventral midbrain neurogenesis in vivo and in human embryonic stem cells. Cell Stem Cell.

[35] Nandy B.S., Mohanty S., Singh M., Behari M., Airan B. (2014). Fibroblast Growth Factor-2 alone as an efficient inducer for differentiation of human bone marrow mesenchymal stem cells into dopaminergic neurons. J. Biomed. Sci..

[36] Chang C.C., Chang K.C., Tsai S.J., Chang H.H., Lin C.P. (2014). Neurogenic differentiation of dental pulp stem cells to neuron-like cells in dopaminergic and motor neuronal inductive media. J. Formos. Med. Assoc..

[37] Briolay A., Lencel P., Bessueille L., Caverzasio J., Buchet R., Magne D. (2013). Autocrine stimulation of osteoblast activity by Wnt5a in response to TNF-alpha in human mesenchymal stem cells. Biochem. Biophys. Res. Commun..

[38] Yu J.M., Kim J.H., Song G.S., Jung J.S. (2006). Increase in proliferation and differentiation of neural progenitor cells isolated from postnatal and adult mice brain by Wnt-3a and Wnt-5a. Mol. Cell. Biochem..

[39] Farías G.G., Alfaro I.E., Cerpa W., Grabowski C.P., Godoy J.A., Bonansco C., Inestrosa N.C. (2009). Wnt-5a/JNK signalling promotes the clustering of PSD-95 in hippocampal neurons. JBC.

[40] Nusse R. (2008). Wnt signaling and stem cell control. Cell Res..

[41] Rosso S.B., Inestrosa N.C. (2013). WNT signalling in neuronal maturation and synaptogenesis. Front. Cell. Neurosci..

[42] Yu Q., Duan Y., Wang Y., Xuan X., Zhou L., Liu W. (2013). Wnt/*β*-catenin signalling regulates neuronal differentiation of mesenchymal stem cells. Biochem. Biophys. Res. Commun..

[43] Cao W., Razanau A., Feng D., Lobo V.G., Xie J. (2012). Control of alternative splicing by forskolin through hnRNP K during neuronal differentiation. Nucleic Acids Res..

