# Supplementary material for: Evaluation of Priming Efficiency of Forskolin in Tissue-Specific Human Mesenchymal Stem Cells into Dopaminergic Neurons: An In Vitro Comparative Study
Source: Cells. 2020 Sep 9;9(9):2058. doi: 10.3390/cells9092058 (PMC7565008; doi:10.3390/cells9092058)
Supplement: Supplementary file 1 [file cells-09-02058-s001.pdf]

Evaluation of Priming Efficiency of Forskolin in Tissue-Specific Human Mesenchymal Stem Cells into Dopaminergic Neurons: An in Vitro Comparative Study

Manisha Singh [1,4], Pardeep Kumar Vaishnav [2], Amit Kumar Dinda [3] and Sujata Mohanty [1,*]

[1] Stem Cell Facility (DBT- Centre of Excellence for Stem Cell Research), All India Institute of Medical Sciences, New Delhi 110029, India; msingh37@jhmi.edu
[2] Electron Microscopy, All India Institute of Medical Sciences, New Delhi 110029, India; pardeepvaishnav@yahoo.com
[3] Department of Pathology, All India Institute of Medical Sciences, New Delhi 110029, India; amit_dinda@aiims.edu
[4] Dr. Solomon H. Snyder Department of Neurosciences, Johns Hopkins University, Baltimore, MA 21218, USA
* Correspondence: drmohantysujata@aiims.edu; Tel: +91-986-839-8194 or +91-981-029-1336

Revival, Expansion, Characterization and Proliferation Studies of human Mesencymal Stem Cells:

MSC Phenotyping:

Cryopreserved BM-MSC, AD- MSC and DP-MSC were used for the study. Informed consent was obtained from the patients or legal representatives at the time of bone marrow, adipose tissue or extracted tooth/teeth collection for previous research projects. Age of patients ranged from 24 to 40 years for both BM-MSC and AD-MSC and 20–28 years for DP-MSC. Cells of 5 healthy donors of each MSC type, which were cryopreserved at first passage were used in this study. BM-MSCs were obtained by direct plating of bone marrow on the cuture dish and AD-MSC and DP-MSCs were obtained by explant culture. No enzymes were used for the extraction (Supplementary Figure 1).

Phenotyping of hMSCs at 3rd passage was performed as per the already standardized protocol of the lab (Nandy et al., 2014). Briefly, hMSCs were harvested using 1X TrypLE Express (Life Technologies, USA) and single cell suspension was prepared in staining buffer at a concentration of 1×10^5 cells/mL. Cells were stained with anti- human CD73-PE, CD90-PECy5, HLA Class I-APC, HLA Class II-FITC (Becton Dickinson, USA), CD29- FITC and CD105- APC (eBioscience, USA). Unlabeled cells were taken as experimental control. The cells were acquired on BD LSR II flow cytometer (Becton Dickinson, USA) with a minimum of 10,000 events for each sample and analyzed with FACs DIVA software (version 6.1.2) (*Supplementary Figure 1*).

Growth Kinetics Assay:

Cell Proliferation Assay (MTT Assay ((3-(4,5-Dimethylthiazol-2-yl)-2,5-diphenyltetrazolium bromide*))*

Cell proliferation rate was assessed on hMSCs at third passage as per the previously reported protocol of the lab (Nandy et al., 2014). Briefly, at 80–90% confluency, cells in T25 culture flasks (Becton Dickinson, USA) were trypsinized using TryplE (Gibco, USA) and harvested by centrifugation at 800 rcf for 5 min. Supernatant was aspirated and pellets were re-suspended in fresh expansion medium. After cell counting, hMSCs from each sample were plated in triplicates at a density of 5×10^6 cells/well in a 96-well plate. Cell expansion media was changed every alternate day. Cell proliferation assay was done on day 1, 3,6,9,12,15 and 22 of the culture, whereexpansion medium was replaced with 180 µl fresh media and 20 µl MTT ((a tetrazole), Sigma, USA) reagent (5 mg/mL in PBS) and incubated for 4 h at 37 ˚C in CO_2 incubator, allowing live cells to form formazan crystals. Post incubation period, media was aspirated and 150 µl DMSO (Sigma, USA) was added to each well in order to dissolve formazan crystals and incubated at 37 ˚C for 30 min in dark. Later, the supernatants were transferred to a separate flat- bottom 96-well plate and the optical densities were obtained at 570 nm and 660 nm using a spectrophotometer (BioTek, USA).

Population Doubling Time (PDT) Assay:

PDT for hMSCs were calculated by plating 1×10^4 cells from 3rd passage per 35 mm petri plate (Becton Dickinson, USA). The assay was performed in triplicates and incubated at 37 ˚C/ 5% CO_2. After obtaining 70–75% confluency, hMSCs were harvested, counted and assessed for viability (Trypan Blue dye exclusion). The PDT was obtained by the formula given below:

$$PDT = \text{T−To Log2(LogN−LogNo)}$$

Where, T: Time of harvesting
To: Time of seeding
N: Number of cells harvested
No: Number of cells seeded

Tri-lineage Differentiation:

hMSCs were characterized by differentiating them into tri-lineage, i.e., cells of osteogenic, chondrogenic and adipogenic lineages as per our previously published research article (Nandy et al., 2014).

Results:

Characterization and proliferation study of hMSCs:

Upon characterizing hMSCs obtained from the three tissue sources, i.e., bone marrow, adipose tissue and dental pulp by surface marker profiling using flow cytometry, it was observed that these hMSCs were positive for CD29, CD73, CD90, CD105 and HLA class I, while they were found negative for hematological markers like CD34/45 and HLA class II, with no significant difference. Trilineage differentiation experiments also supported the results obtained from surface marker profiling, with exception of DP- MSCs showing low levels of differentiation into adipocytes (Supplementary Figure 1).

Proliferation rate (MTT assay) and population doubling time (PDT) of all the three hMSC types were also studied. It was observed that the PDT of DP- MSCs (36 ± 3.5 h) was significantly lower than that of BM-MSCs (40 ± 2.8 h) or AD- MSCs (40.90 ± 0.5686 h). These results were further strengthened by proliferation assay of the three hMSC types under study (Supplementary Figure 2).

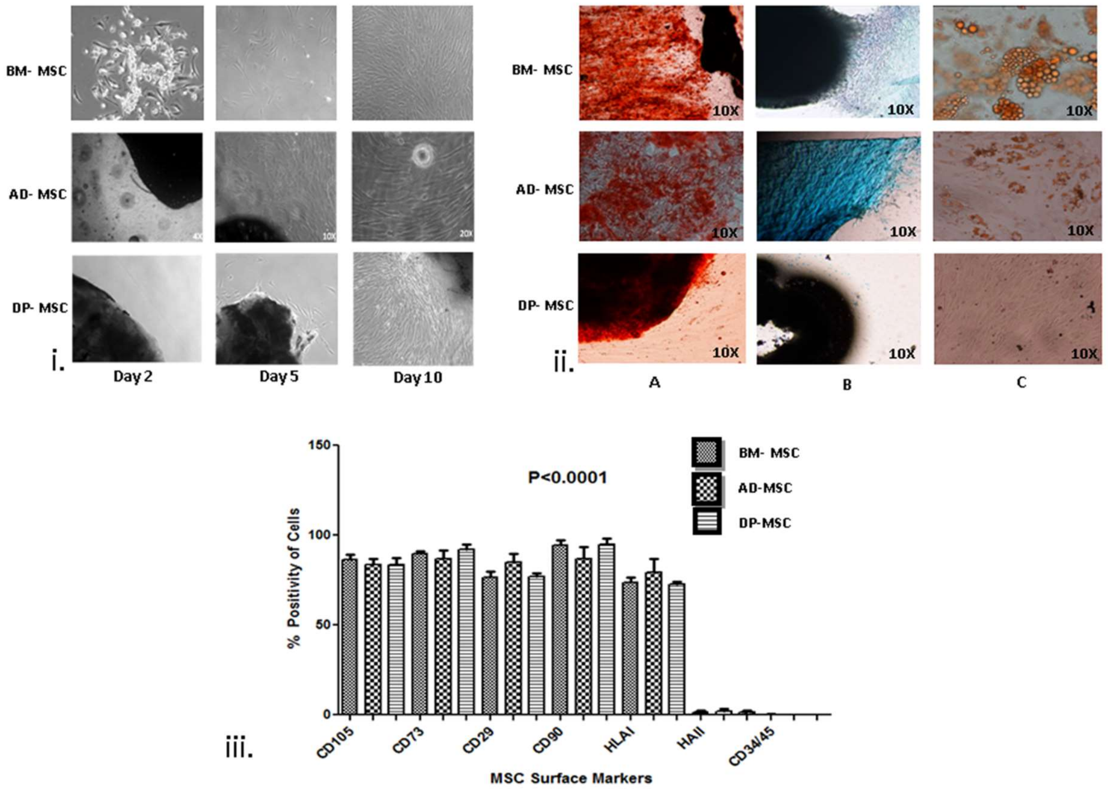

Supplementary Figure 1: Isolation, expansion, characterization of hMSCs. i) Explant culture method to isolate MSCs from bone marrow, adipose tissue and dental pulp; ii) Tri- lineage differentiation of hMSCs into A. Osteocytes, B. Chondrocytes and C. Adipocytes; iii) Surface marker profiling of hMSCs by flow cytometry.

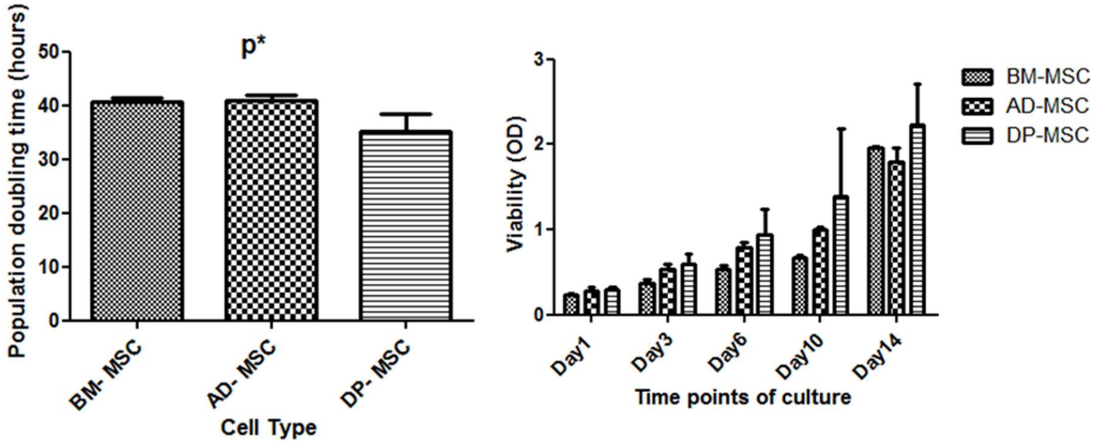

Supplementary Figure 2: Proliferation assay of hMSCs i) Population doubling time of hMSCs; ii) Proliferation assay of hMSCs by MTT assay

Reference:

Nandy, S. B.; Mohanty, S.; Singh, M.; Behari, M.; Airan, B. Fibroblast Growth Factor-2 alone as an efficient inducer for differentiation of human bone marrow mesenchymal stem cells into dopaminergic neurons. J. of Biom. Sci. **2014**, *21(1)*, 83, doi: 10.1186/s12929-014-0083-1.

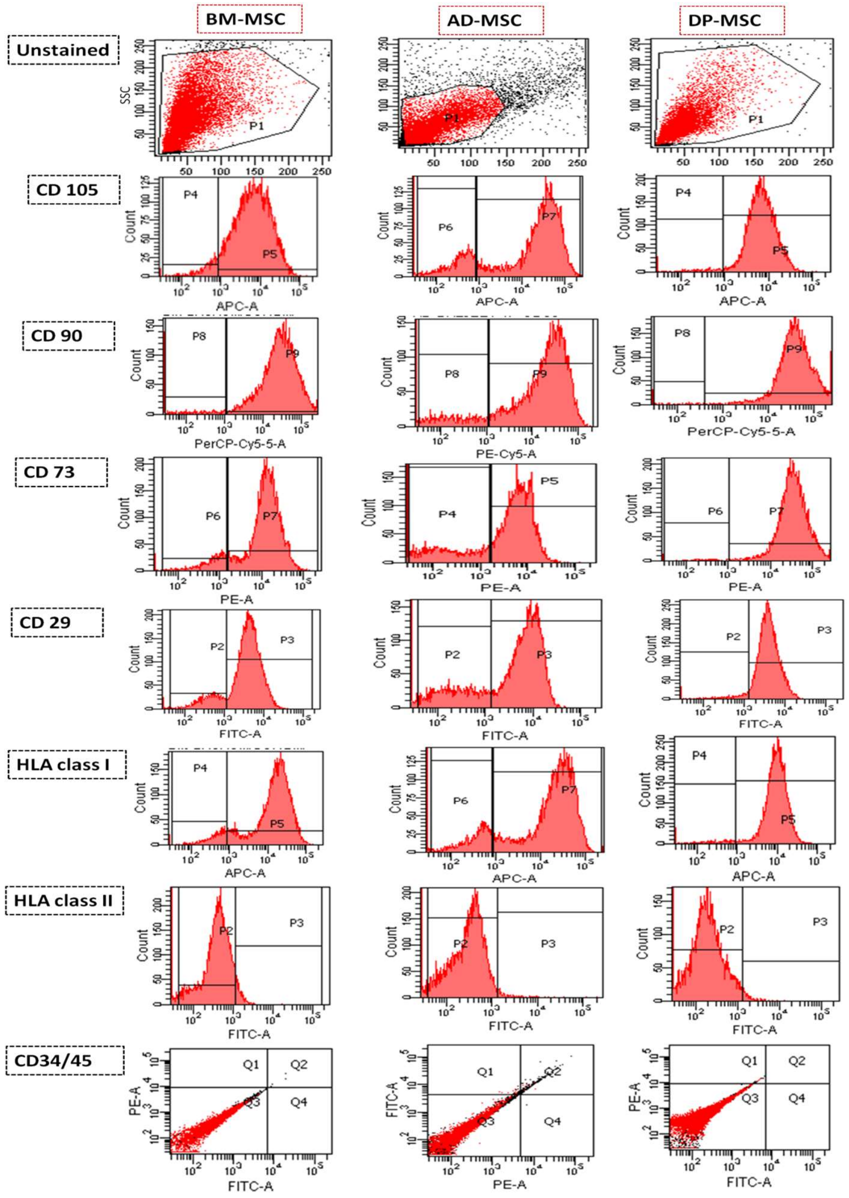

Supplementary Figure 3: Representative Graphs of surface marker profiling of hMSCs

Original blots used in the manuscript

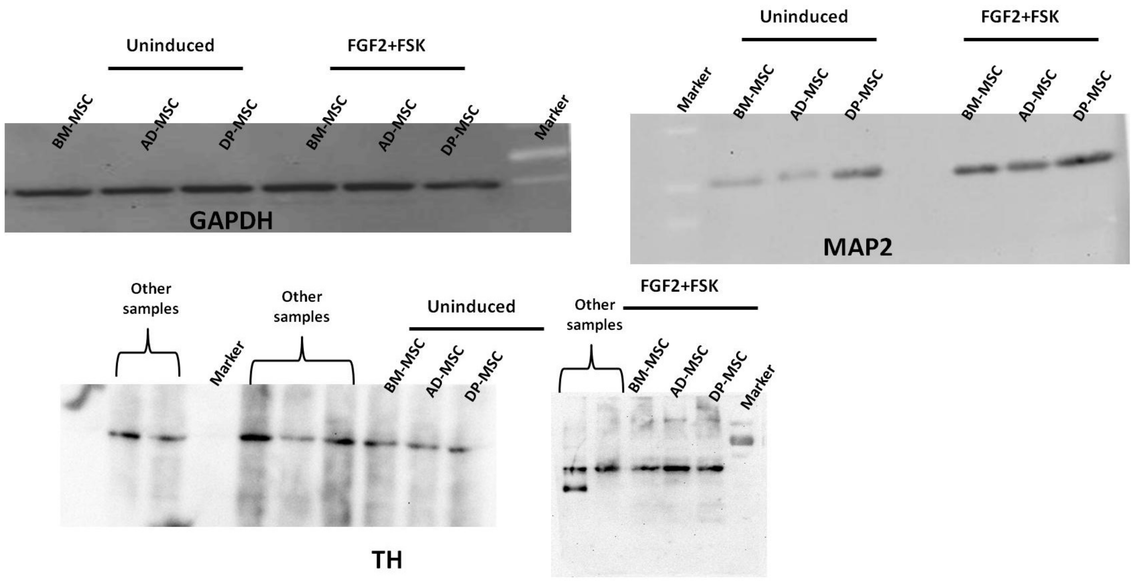

Supplementary Figure 4: Original blots of western blotting blots used in the study.
